# Green Light Improves Photosystem Stoichiometry in Cucumber Seedlings (*Cucumis sativus*) Compared to Monochromatic Red Light

**DOI:** 10.3390/plants10050824

**Published:** 2021-04-21

**Authors:** Nicholas B. Claypool, J. Heinrich Lieth

**Affiliations:** Plant Sciences, University of California, Davis, CA 95616, USA; nbclaypool@gmail.com

**Keywords:** blue light, *Cucumis sativus L.* (cucumber), green light, light-emitting diode (LED), morphology, photosynthesis, red light, intrinsic water use efficiency (iWUE), photostationary state of phytochrome (PSS), photosynthetic photon flux density (PPFD), yield photon flux (YPF)

## Abstract

It has been shown that monochromatic red and blue light influence photosynthesis and morphology in cucumber. It is less clear how green light impacts photosynthetic performance or morphology, either alone or in concert with other wavelengths. In this study, cucumber (*Cucumis sativus*) was grown under monochromatic blue, green, and red light, dichromatic blue–green, red–blue, and red–green light, as well as light containing red, green, and blue wavelengths, with or without supplemental far-red light. Photosynthetic data collected under treatment spectra at light-limiting conditions showed that both red and green light enhance photosynthesis. However, photosynthetic data collected with a 90% red, 10% blue, 1000 µmol photons m^−2^ s^−1^, saturating light show significantly lower photosynthesis in the green, red, and red–green treatments, indicating a blue light enhancement due to photosystem stoichiometric differences. The red–green and green light treatments show improved photosynthetic capacity relative to red light, indicating partial remediation by green light. Despite a lower quantum efficiency and the lowest ambient photosynthesis levels, the monochromatic blue treatment produced among the tallest, most massive plants with the greatest leaf area and thickest stems.

## 1. Introduction

While light provides energy for photosynthesis, it also directs how plants grow through the use of photoreceptors, such as phytochrome and cryptochrome, which allow the plant to respond to changes in spectral quality ranging from ultraviolet to far-red wavelengths [1]. These responses have implications for plant growth in natural conditions, from the forest floor to field conditions, as well as artificial environments such as indoor agriculture illuminated entirely by electrical lighting [2,3,4,5].

Already in the 1970s, research showed that various wavelengths of light had differing effects on photosynthesis on a quantum yield basis [6,7,8,9]. In particular, red and blue wavelengths were shown to result in greater rates of photosynthesis than green wavelengths. More recently, studies have examined chemical and structural changes to photosystem stoichiometry and function as they relate to photosynthesis [10,11]. It has been found that monochromatic red light results in poor growth characterized by a low photosynthetic capacity, unresponsive stomatal conductance, low specific leaf weight (leaf mass divided by leaf area), and low maximum quantum efficiency of photosystem II [10,11,12,13]. However, the addition of blue light can ameliorate these negative responses, restoring photosynthetic and physiological characteristics comparable to plants grown under white light [13]. In addition to photosynthetic responses, there is widespread interest in how spectral quality changes other aspects of physiology and development.

One commonly reported morphological response is specific leaf weight (SLW), also called leaf mass area (LMA), which is the mass of a leaf divided by its area. This is because SLW represents an investment by the plant per unit of leaf area created, so that plants with the same plant-level net photosynthesis could have very different leaf area due to differences in SLW and different net photosynthesis rates per unit leaf area. Previous studies have found that SLW tends to increase with increasing proportion of blue light [10,14,15,16].

Many studies have focused on the role of red, blue, and combinations of red and blue light [10,11,15,17,18,19]. Comparatively little research has been done on green light [20,21,22]. Nevertheless, it is important to include green light in spectral quality studies, as physiological responses can be the result of interactions between different wavelengths as well as other environmental variables. Green light pulses inhibited blue light-induced phototropism in dark-grown seedlings while enhancing blue light-induced phototropism in light-grown seedlings [23]. Earlier studies showed that green light reversed blue light-induced stomatal opening [24,25].

The present study used cucumber as a model plant for several reasons. First, cucumber has been documented to have high sensitivity to light quality [14,15,16,26,27]. Second, cucumber is one of the most produced crops under protected cultivation globally under artificial and supplemental lighting systems [28]. Third, while responses to red and blue light have been studied somewhat extensively in cucumber, to our knowledge, the response to green light and interactions between blue, green, and red light is less well understood [11,15,16,29].

Our objective was to characterize cucumber photosynthetic adaptation to diverse spectra containing combinations of red, green, and blue light to determine how light signals in complex spectra interact to influence photosynthesis. Additionally, we sought to understand how photosynthetic differences influence biomass accumulation and morphology.

## 2. Results

### 2.1. Photosynthesis

**Figure 1:**
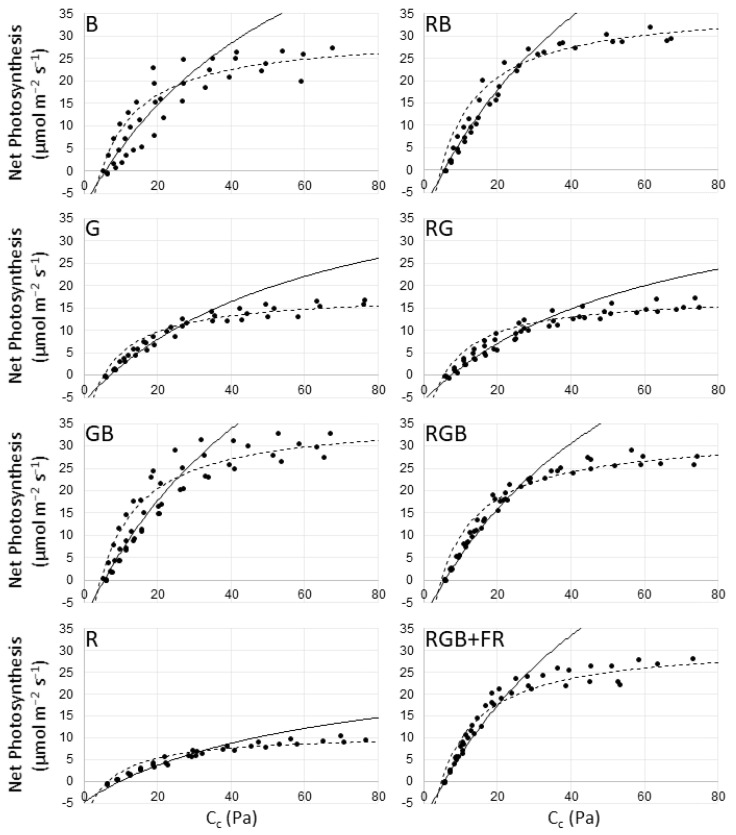
Net photosynthesis (A) vs. cellular CO_2_ concentration (C_c_) curve fitting for each light treatment. Filled circles represent observed net photosynthesis (A) relative to calculated Cc values. The solid line shows Rubisco limitation, while the dotted line fits RuBP limitation. Triose-phosphate utilization (TPU) limitation was not apparent.

Fitting net photosynthesis (A) vs. cellular CO_2_ concentration (C_c_) curves to net photosynthesis over a range of CO_2_ concentrations allows for the estimation of parameters that relate to leaf-level photosynthesis and the underlying biochemistry limiting photosynthetic assimilation of CO_2_ (Figure 1), with estimates for the potential electron transport rate (J) and maximum RuBP carboxylation rate (V_cmax_) in Table 1. A C_c_ value of ~270 ppm corresponded to ambient concentrations of CO_2_ of 400 ppm under measurement conditions. At that level, photosynthesis was highest in RB and GB (23.9 and 23.6 µmol CO_2_ m^−2^ s^−1^, respectively) followed by RGB, RGB + FR, and B (21.0, 20.5, and 19.6 µmol CO_2_ m^−2^ s^−1^, respectively). Considerably lower photosynthesis values are found for G and RG (11.3 and 10.2 µmol CO_2_ m^−2^ s^−1^, respectively) with R having the lowest photosynthesis of all groups at 5.8 µmol CO_2_ m^−2^ s^−1^.

The estimated maximum rate of Rubisco carboxylation is significantly higher in RB and RGB + FR than all other treatments except GB. The G, R, and RG treatments have significantly lower estimates for V_cmax_ and J than treatments containing blue light—the B, GB, RB, RGB, and RGB + FR treatments (Table 1).

**Figure 2:**
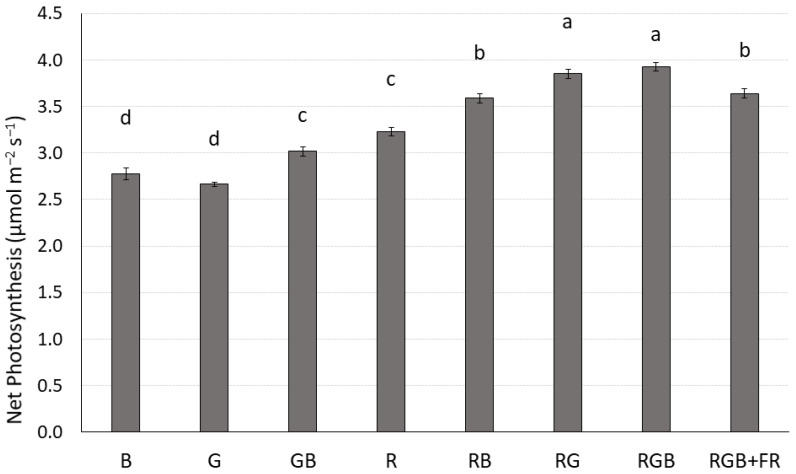
Net photosynthesis (A) under ambient treatment lighting. Different lowercase letters indicate significant differences (*p* ≤ 0.05; *n* = 6). Error bars are the standard error. Uppercase letters indicate light treatments.

The photosynthesis measurements under 1000 µmol photons m^−2^ s^−1^, saturating light differ substantially from the photosynthesis measurements under ambient, treatment light at 170 µmol photons m^−2^ s^−1^ (Figure 2). Under ambient conditions, net photosynthesis was highest in RGB and RG, followed by RB and RGB + FR which had significantly higher net photosynthesis than GB or R. The B and G treatments had the lowest net photosynthesis under ambient conditions.

Overall, there was no correlation between net photosynthesis under ambient, treatment lighting at 170 µmol photons m^−2^ s^−1^ and those observed at the same CO_2_ concentration under saturating 90% red, 10% blue light at 1000 µmol photons m^−2^ s^−1^.

### 2.2. Chlorophyll Fluorescence

The relative operating efficiency of PSII was highest in the GB treatment and lowest in the R treatment (Table 2). The R and RG treatments had significantly higher ΦPSII values than the R treatment, but significantly lower than all treatments containing blue light. The maximum quantum efficiency of PSII photochemistry (F_v_/F_m_) was slightly under 0.83—indicating mild stress—for all treatments with no significant differences observed for any treatments (Table 2). Both light-induced and non-light-induced nonphotochemical quenching were higher in treatments lacking blue light (R, G, and RG) compared to treatments containing blue light (B, RB, GB, RGB, and RGB + FR).

Under light-saturating conditions, net photosynthesis was significantly lower in the G, R, and RG treatments than all other treatments (Figure 1). However, this had no apparent effect on photosynthesis under ambient conditions (Figure 2). While ambient photosynthesis was lowest in the G treatment, net photosynthesis in R was comparable with GB, and significantly higher than B or G. Finally, ambient photosynthesis in RG was significantly higher than all other treatments save RGB (Figure 2).

### 2.3. Shoot Characteristics

**Figure 3:**
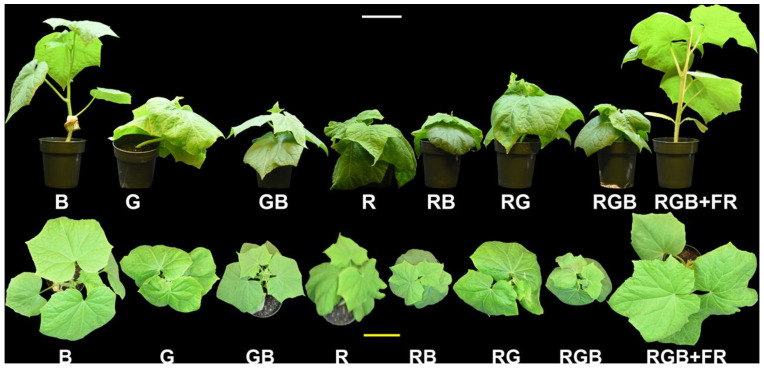
Profile and overhead images of representative plants, chosen by selecting the plant that was closest in dry weight and height to the treatment average. This image is a composite to allow for visual comparison between treatments. Cucumber seedlings were grown under blue (B), green (G), green–blue (GB), red (R), red–blue (RB), red–green (RG), red–green–blue (RGB), and red–green–blue with far-red (RGB + FR) light. The white bar in the upper middle is 10 cm for profile images, while the yellow bar in the lower middle is 10 cm for overhead images.

**Figure 4:**
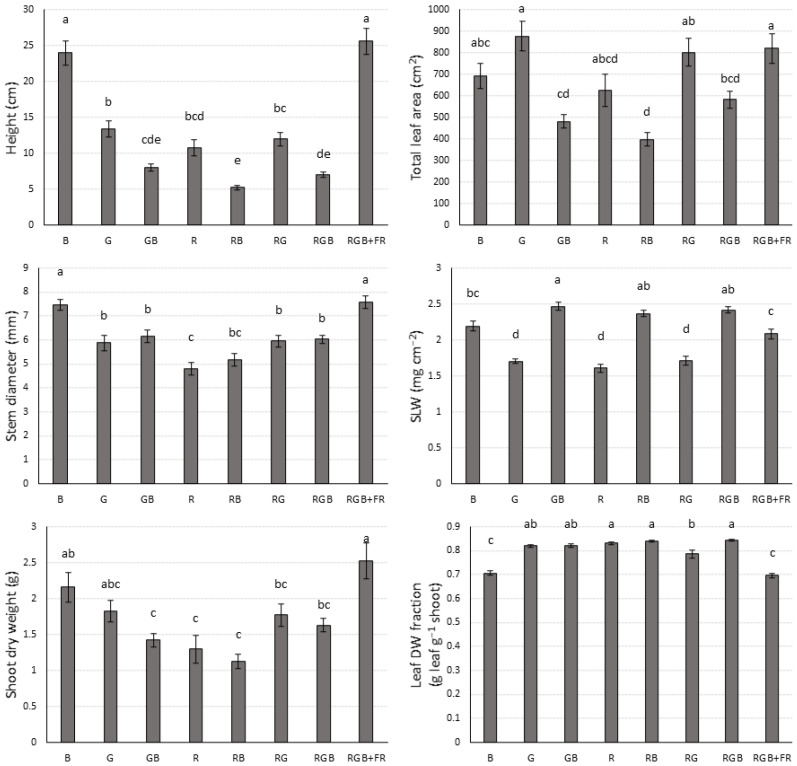
Shoot characteristics of cucumber under diverse spectra. Different lowercase letters indicate significant differences (*p* ≤ 0.05; *n* is between 25 and 30 for each treatment). Uppercase letters indicate light treatments.

Qualitative differences between treatments can be seen in Figure 3, which shows exemplar plants (those closest to treatment average in height and mass) from the replication experiment for each treatment. Shoot dry weight showed no clear trends, except that far-red light increased shoot dry weight, with an average of 2.53 g per plant for the RGB + FR treatment and only 1.63 g for the RGB treatment (Figure 4). The RGB + FR and B treatments had significantly higher dry weight than the GB, R, and RB treatments, while the RGB + FR treatment also had significantly higher dry weight than the RG and RGB treatments (Figure 4).

Far-red light also increased plant height, with the RGB + FR treatment being significantly taller than the RGB treatment (Figure 4). Conversely, supplemental blue light decreased plant height, with shorter plants in RB than R, GB than G, and RGB than RG. However, plants grown in the B treatment were taller than all other treatments except RGB + FR. The RGB + FR and B treatments, in addition to being the tallest, also had the lowest leaf dry weight fraction (leaf dry weight divided by shoot dry weight) (Figure 4).

There were no clear trends for stem diameter, except that far-red light enhanced stem diameter, with plants in the RGB + FR treatment having significantly greater stem diameter than plants in the RGB treatment (Figure 4).

The RGB + FR treatment also resulted in significantly greater leaf area than the RGB treatment (Figure 4). Due to high within groups variability, there were no other significant trends in leaf area, although with a larger sample size, a trend of decreasing leaf area with supplemental blue light may be observed.

Specific leaf weight (SLW), the dry weight of a leaf divided by its area, does show a clear trend with blue light significantly increasing SLW. Higher specific leaf weights were observed in the GB treatment relative to G, RB relative to R, and RGB relative to RG (Figure 4). Far-red light decreased SLW, with the RGB + FR treatment having significantly lower SLW than the RGB treatment.

### 2.4. Stomatal Characteristics

**Figure 5:**
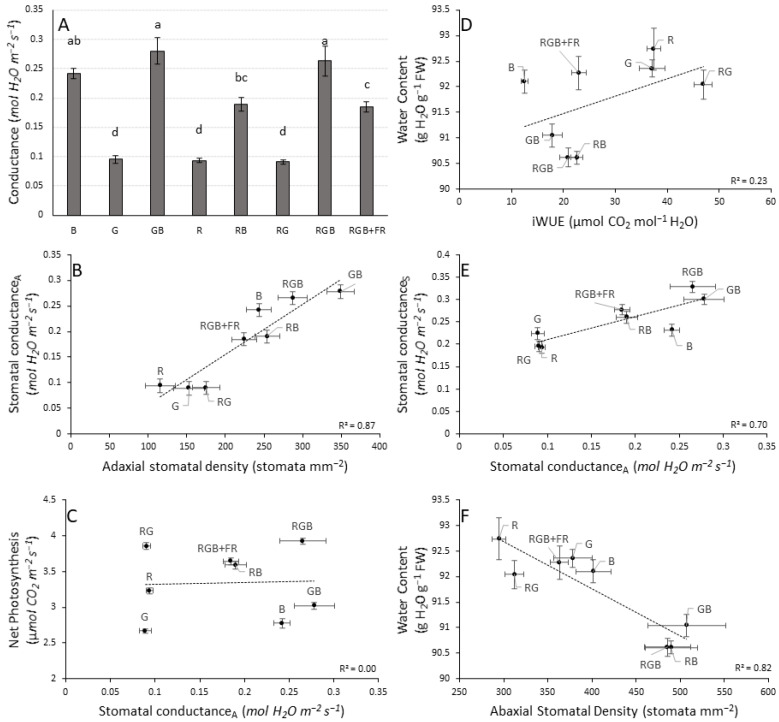
(**A**): Stomatal conductance under saturating light by light treatment. Different lowercase letters indicate significant differences (*p* ≤ 0.05; *n* is between 25 and 30 for each treatment). (**B**): Stomatal conductance under ambient, treatment lighting (Stomatal conductance_A_) vs. adaxial stomatal density. (**C**): Net photosynthesis under ambient, treatment lighting vs. stomatal conductance under ambient, treatment lighting. (**D**): Water content vs. instantaneous water use efficiency. (**E**): Stomatal conductance under saturating light vs. stomatal conductance under ambient, treatment lighting. (**F**): Water content vs. abaxial stomatal density. Uppercase letters indicate light treatments.

Stomatal conductance under ambient lighting was significantly higher in B (0.24 mol m^−2^ s^−1^) relative to R (0.09 mol m^−2^ s^−1^) or G (0.09 mol m^−2^ s^−1^) and significantly higher in GB (0.28 mol m^−2^ s^−1^) relative to G, in RB (0.19 mol m^−2^ s^−1^) relative to R, and in RGB (0.27 mol m^−2^ s^−1^) relative to RG (0.09 mol m^−2^ s^−1^), demonstrating a blue light-mediated increase in stomatal conductance (Figure 5A).

We found a significant increase in conductance from RB to RGB, but there was no difference in stomatal conductance between G, R, and the RG treatments (Figure 5A). Far-red light decreased stomatal conductance, with conductance significantly lower in RGB + FR (0.18 mol m^−2^ s^−1^) compared to RGB. Stomatal conductance under ambient, treatment lighting was highly correlated with adaxial stomatal density (Figure 5B, R^2^ = 0.87). However, stomatal conductance was not correlated to net photosynthesis under ambient, treatment lighting (Figure 5C, R^2^ = 0.00).

When measuring the A vs. C_c_ curves, all plants were subjected to saturating levels of 90% red, 10% blue light at 1000 µmol photons m^−2^ s^−1^. Despite being illuminated with the same spectrum, conductance trends were similar to those obtained when illuminated by treatment spectra. Overall, average conductance values under saturating light were higher in all treatments compared to ambient lighting conditions except the B treatment, with R^2^ = 0.70 (*p* < 0.01) (Figure 5E).

Like conductance, blue light resulted in an increased stomatal density, with abaxial stomatal density higher in B relative to R (although not different from G), and higher abaxial density in GB relative to G, RB relative to R, and RGB relative to RG. The same trends were found for adaxial stomatal density, except that adaxial stomatal density in B was significantly higher than G (Table 3).

Abaxial stomatal density was also lower in RGB + FR than RGB, though there was no difference in adaxial stomatal density (Table 3). We observed significantly higher abaxial:adaxial ratios for the G and R treatments relative to all other treatments.

Intrinsic water use efficiency (iWUE) under ambient conditions, which is calculated by dividing the net photosynthesis by stomatal conductance, was highest in RG, followed by G and R, while iWUE was lowest in the B treatment. Like [30], we found no difference in iWUE between RB and RGB; however, [31] did find a significant increase in iWUE in a low R:FR treatment compared to a high R:FR treatment, while we found no difference between the RGB and RGB + FR treatment.

Intrinsic water use efficiency had only a weak correlation with water content at harvest (Figure 5D, R^2^ = 0.23). Water content, the percentage of fresh weight from water, was lower in GB, RB, and RGB than all other treatments. Interestingly, while stomatal conductance was best explained by adaxial stomatal density, water content at harvest was best explained by abaxial stomatal density (R^2^ = 0.82, Figure 5F).

## 3. Discussion

Previous experiments found that cucumber measured under saturating light and grown under monochromatic red light showed lower photosynthesis than cucumber plants grown under red–blue light, consistent with our findings [10].

The J and V_cmax_ values calculated suggest that blue light significantly enhances photosynthetic capacity relative to treatments lacking blue light. Since these values are lowest in the R treatment, and significantly higher in the RG and G treatments, we can also conclude that green light improves photosynthetic capacity relative to monochromatic red light. However, because the RG and G treatments have significantly lower J and V_cmax_ values than treatments containing blue light, the effect of green light must be lesser than that of blue light.

Previously, calculations of V_cmax_ and J were found to be significantly higher in B than R; however, they found no difference in V_cmax_ between R and RB, while estimates for V_cmax_ were significantly higher in RB than R in our experiment [12]. Others calculated V_cmax_ for a low R:FR treatment as significantly lower than for a high R:FR treatment, while we found V_cmax_ to be significantly higher in the RGB + FR treatment compared to the RGB treatment [31].

Our findings suggest that green light enhanced net photosynthesis, since values for V_cmax_, J, and net photosynthesis were significantly greater in GB relative to B, RG relative to R, and RGB relative to RB (Figure 3). Photosynthesis was also significantly higher in RB relative to B, RG relative to G, and RGB relative to GB, suggesting a red light enhancement. Others have found that a broader spectrum resulted in higher fixation than a red–blue light treatment for tomato and poinsettia but saw no difference in cucumber at ambient CO_2_ concentrations [32]. They did observe a difference at elevated CO_2_, similar to the enhancement we saw for photosynthesis in RGB relative to RB at ambient CO_2_ and light levels. Others have found higher fixation in B than R for cucumber while we observed the opposite under ambient conditions [11,12,15,33]. During A vs. C_c_ measurements, plants from the B treatment had a higher net photosynthesis level than plants in the R treatment, indicating that the choice of spectral composition, intensity, or both is critically important to comparing net photosynthesis levels between treatments, even when the same light source is used for each treatment.

It is possible that the red and green light enhancement can be described in part by the ‘enhancement effect’ or ‘Emerson effect’ which refers to the phenomenon where photosynthesis from combined spectra can be greater than the sum of its parts due to excitation energy distribution between photosystem I and photosystem II [34,35,36].

### 3.1. Chlorophyll Fluorescence

The maximum quantum efficiency of PSII photochemistry (F_v_/F_m_) indicates how effectively PSII uses absorbed light energy to reduce the primary quinone acceptor of PSII (Q_A_) [37]. In practice, this measure can be used to assess stress in plants, as a value of ~ 0.83 is very consistent across species in non-stressed leaves [38]. Values below 0.83 indicate stress and a reduced maximum photosynthetic capacity; however, photosynthesis may not be reduced under ambient conditions as the quantum yield of PSII (ΦPSII) is generally considerably lower than F_v_/F_m_, especially under high light intensity. A low F_v_/F_m_ is one of the symptoms of red light syndrome [11,12,13].

F_v_/F_m_ was qualitatively lower in G, R, and RG than all other treatments, indicating a reduced maximum photosynthetic efficiency with values suggesting mild stress (Table 4). These qualitative differences are supported by previous findings that a comparatively higher level of blue light in LED treatments increased F_v_/F_m_ relative to high pressure sodium treatments [39]. Others have also concluded that blue light enhances PSII photochemistry relative to red light [11,12,13].

PSII operating efficiency decreases with increasing light intensity, primarily due to a reduced ability to oxidize Q_A_ rather than an increase in non-photochemical quenching (NPQ) [37,40]. Therefore, it is not surprising that under saturating light the PSII operating efficiencies observed were much lower than F_v_/F_m_. PSII operating efficiency was significantly lower in G, R, and RG than all other treatments (with ΦPSII in R significantly lower than RG, which was significantly lower than ΦPSII in G) (Table 2). It is not possible to estimate electron transport rate or the quantum yield of CO_2_, since we cannot account for alternative electron sinks to PSII because these measurements were taken under atmospheric O_2_ concentrations. Nevertheless, ΦPSII gives an estimate on the upper limit of possible photosynthetic carbon assimilation under a given condition, and the trend observed is very similar to the trend in net photosynthesis observed.

Despite the lower ΦPSII values, ΦNPQ, the quantum yield of light-induced quenching, and ΦNO, non-light-induced quenching, are both significantly higher in the G, R, and RG treatments than all other treatments (Table 2). Together, these data suggest that electron acceptors downstream of PSII are insufficient in the G, R, and RG treatments compared to the other treatments, and that G, R, and RG treatments are compensating by increasing nonphotochemical quenching to reduce photo-induced damage. Since the spectral quality and intensity used to excite the photosystems were identical across treatments during light-saturated measurements, one would expect differences in net photosynthesis and chlorophyll fluorescence to be related to adaptive differences between light treatments.

Plants have a variety of mechanisms to respond to changes in light quality. In the short term, light-harvesting complex II (LHC-II) can be transferred from PSII to PSI to help balance excitation energy between the two systems to improve electron transport efficiency [41]. In the long term, algae, cyanobacteria, and higher plants adjust the stoichiometry of photosystem I (PSI) and photosystem II (PSII) in response to light quality to improve photosynthetic efficiency [42,43,44,45] as well as their pigment composition [42,46] to more efficiently absorb ambient light.

PSII is primarily excited by wavelengths at ~450–640 nm while PSI uses light above 680 nm much more efficiently than PSII [47]. Since the blue LEDs are the only source of photons at 450 nm in our light treatments, treatments lacking these wavelengths (G, R, and RG) likely have adjusted stoichiometry to decrease the number of PSI complexes relative to PSII to improve electron transport efficiency due to less efficient excitation of PSII relative to PSI. As the green LEDs supply light within the range that PSII can use effectively, this stoichiometric adjustment would be expected to be most pronounced in the R treatment, and less so in the G and RG treatments.

When the plants were exposed to the novel light treatment (90% red, 10% blue) during A vs. C_c_ measurements they could use transient LHC-II to improve the balance of excitation between PSI and PSII, but the capacity to balance in the G, R, and RG treatments may have been limited by extreme stoichiometric differences not seen in the other treatments. This would explain the much poorer performance of these three treatments relative to the other treatments and the poorer performance of R relative to G and RG during A vs. C_c_ measurements, while the same long-term adaptations may have allowed for the trends seen in Figure 1 during measurement under ambient lighting.

In any case, neither the photosynthesis measurements under saturating 90% red, 10% blue light at 1000 µmol photons m^−2^ s^−1^ nor ambient light at 170 µmol photons m^−2^ s^−1^ correlate well with shoot dry weight. There are many potential reasons for the lack of correlation between shoot biomass and photosynthesis measurements. First, the dry weight data include only shoot biomass, not root biomass. It is possible that with root biomass, the whole-plant biomass values would correlate well with the net photosynthesis measurements observed. The photosynthesis values presented are on a per-area basis. Leaf area and specific leaf weight (leaf area divided by leaf dry weight) vary between treatments. It is therefore possible that plants with equivalent net photosynthesis rates could have very different whole plant growth rates due to differences in leaf area [48].

### 3.2. Stomatal Conductance

Others have also noted a trend of increasing stomatal conductance in cucumber with increasing blue light [11,15]. Significantly higher stomatal conductance in cucumber grown under monochromatic blue light compared to cucumber grown under monochromatic red light was found here and previously [33]. Another study also measured significantly higher stomatal conductance in cucumber seedlings under a monochromatic blue light treatment relative to a monochromatic red light treatment and found no difference between the B treatment and RB treatment, which our results support [12]. However, we found conductance in RB to be significantly greater compared to R, while they found no difference in conductance between the R and RB treatment. Our results differ from a finding that there was no difference in stomatal conductance of cucumber between a white light LED treatment and a red–blue light treatment, while we found conductance to be significantly higher in RGB compared to RB [30]. It is possible that this is because our RGB treatment was roughly 2:1:2 B:G:R light while the white light treatment in their experiment was roughly 1:2:1 B:G:R light. In Arabidopsis, red–blue light increased stomatal aperture more than red light alone, while red–green–blue light showed no increase in aperture relative to red light alone [24].

Our findings on blue and green light effects on stomatal conductance are similar to those findings in Arabidopsis, as they observed no difference in aperture between R and RG. However, they found a significant decrease in stomatal aperture for monochromatic G compared to R or RG while we found no difference. It is possible that this is because our plants had time to form long-term adaptations to light quality, while the Arabidopsis leaves were being exposed to a novel lighting condition and therefore only had short-term responses.

Another study found that a decreased ratio of R:FR resulted in a decrease in stomatal conductance in cucumber relative to a treatment with high R:FR light [31]. This is similar to our findings between the RGB and RGB + FR treatments, where RGB + FR had significantly lower conductance than the RGB treatment.

The fact that similar conductance trends were observed despite illumination under very different spectral quality suggests that the results are driven more by physical differences in the leaves than transient chemical expression induced by light signaling.

This is supported by the stomatal density data shown in Table 3 and the correlation between stomatal conductance under ambient lighting and adaxial stomatal density (Figure 5).

The stomatal density trends we observed agree with previous findings of significantly higher average stomatal density in B and RB light treatments relative to R [29]. Others found that a decreased R:FR ratio resulted in a decreased stomatal density, although they found a significant decrease in adaxial rather than abaxial stomatal density [31]. They also found a significant difference in the abaxial to adaxial stomatal ratio (AB:AD) while we found no difference between RGB and RGB + FR.

Stomatal density also explains more than half of the variation found in intrinsic water use efficiency (iWUE) under ambient conditions, which is calculated by dividing the net photosynthesis by stomatal conductance (R^2^ = 0.58, Figure 5).

### 3.3. Morphology

In red–blue light treatments, increasing proportions of blue light led to reduced leaf area [15]. Previously, it was found that monochromatic blue light resulted in the highest leaf area, followed by white light, which had significantly higher leaf area than monochromatic red light [33]. We did not find any statistically significant differences between these treatments, but that may be due to the high within-group variation and relatively low sample size, as the mean leaf areas in our treatments follow the same trend. The same study found no difference between R, B, and RB treatments, while we found RB to have significantly lower leaf area than R or B [33]. The effect of decreasing the R:FR ratio on leaf area is species specific, with some showing decreased leaf area, while others like petunia show an increase in leaf area [49]. In our experiment, far-red light mediated increased leaf area, which has been found by others [50].

Cucumber grown under monochromatic red light was taller than cucumber grown under a 1:1 ratio of red to blue, with both treatments being shorter than a monochromatic blue treatment, which is consistent with our findings [15]. Far-red-regulated increases in plant height are well documented [50,51,52,53]. As the height of the light fixtures was not adjusted during the experiment, it is possible that plants which grew taller received more irradiation than shorter plants, potentially affecting total shoot biomass.

The leaf area and specific leaf weights observed may also help to explain the average shoot biomass for each treatment. For example, the G treatment had the lowest net photosynthesis per unit leaf area along with the B treatment (Figure 2). However, both the G and B treatments had high leaf area, potentially allowing for the same or greater total photosynthesis as a treatment with lower leaf area but higher net photosynthesis per unit area, such as the RB treatment which had higher net photosynthesis per unit area, but lower leaf area and shoot dry weight.

In the case of the G treatment, a low SLW meant that more leaf area could be produced using the same amount of photosynthate compared to the RB treatment. The SLWs for the B and RB treatments were not significantly different, but the B treatment plants were much taller, potentially resulting in higher average light intensity for the duration of the experiment.

## 4. Materials and Methods

The experimental work consisted of 2 replications over time. Cucumber (*Cucumis sativus* cv. Diva) seeds were germinated in total darkness at 32 °C. Once germinated, seeds were transplanted into 4 in. pots containing UC mix (⅓ peat moss, ⅓ redwood sawdust, ⅓ fine sand), covered with an additional 100 cm^3^ of UC mix, and randomly distributed into their light treatment chambers. Plants were irrigated with ½ strength Hoagland’s solution every third day for the first two weeks, then daily thereafter [54]. Leaf photosynthetic rate, stomatal conductance, and fluorescence measurements were obtained during both replications. Morphological measurements were made four weeks after transplant.

In both replications, plants were grown in chambers 61 cm wide, 122 cm long, and 90 cm tall. An 8 in. duct fan exhausted air from the chambers so that the average temperature was 23.0 ± 0.2 °C when the lights were on and 20.9 ± 0.2 °C when the lights were off.

**Figure 6:**
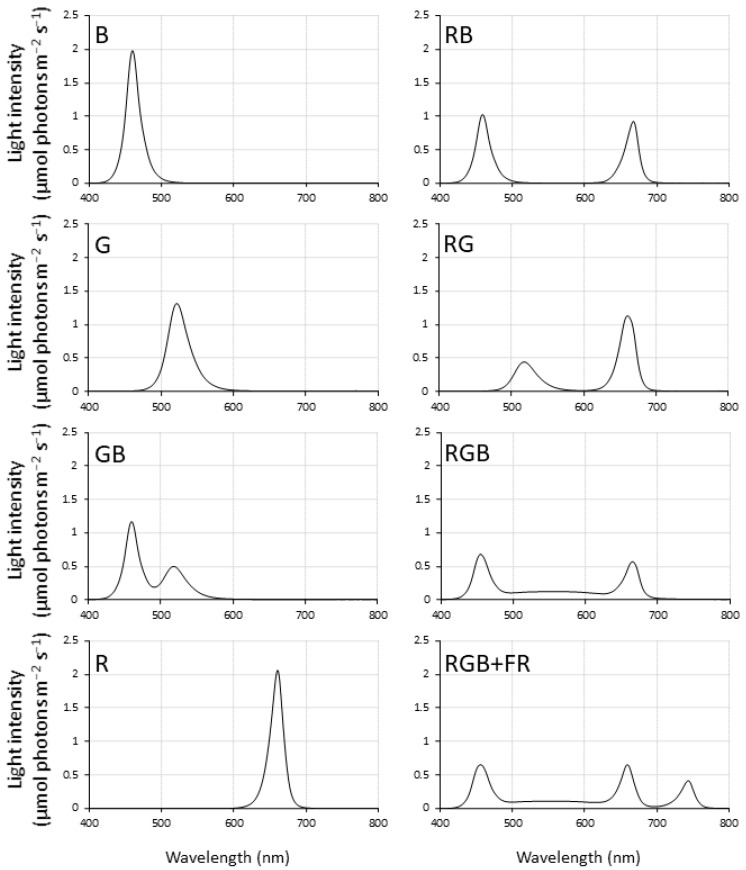
Light treatment spectra: monochromatic blue (B), monochromatic green (G), monochromatic red (R), green–blue (GB), red–blue (RB), red–green (RG), red–green–blue (RGB), and red–green–blue with far red (RGB + FR).

Each chamber was illuminated with lamps consisting of various light-emitting diode (LED) bars (Demegrow, Inc., Sacramento, CA, USA) specifically designed to provide a custom spectrum in each chamber. Fixtures consisted of different combinations of diodes emitting far-red, red, green, or blue light, with peak intensities at wavelengths of 744 nm, 661 nm, 521 nm, and 460 nm, respectively. Spectra of the resulting lamp systems were measured with a JAZ spectrometer (model: JAZ spectrometer, Ocean Optics, Largo, FL, USA). The full width at half maximum for each peak was 21.8, 20.7, 33.6, and 21.6 nm for far-red, red, green, and blue peaks, respectively (Figure 6). Green light in the RGB and RGB + FR treatments came from 15,000 K white LEDs, which is why the green peak is broader in these treatments than other treatments containing green light. Each fixture installation was configured so that all had comparable photosynthetic photon flux density (PPFD) levels of 120 μmol photons m^−2^ s^−1^ in each chamber. This was achieved by raising or lowering the lamp array in each chamber and averaging measurements over a 45-point grid.

Where both colors were present, the intensity of blue and red are roughly 1:1, blue and green are 2:1, and red and green are 2:1; actual percentages of total light as in Table 4. Since the energy of far-red light does not contribute to photosynthetic photon flux density (PPFD), the RGB and RGB + FR treatments have roughly the same PPFD and light ratios between 400 and 700 nm, but 18% of all incident irradiation between 400 to 800 nm in the RGB + FR treatment was in the far-red (700 to 800 nm) region. One percent of incident photons were in the far-red region in the RGB treatment, while all other treatments had negligible levels of far-red light. In addition to the traditional color quantification (red 600–700 nm, green 500–600 nm, and blue 400–500 nm), the light is reported based on the quantity from each ‘color’ of LED bar. This was determined by only powering LED bars of a given light color and measuring PPFD, then calculating the percentage of total PPFD from that bar color (Table 4).

Yield photon flux (YPF) was calculated for each light treatment according to [9] by multiplying relative quantum efficiency at a given wavelength with the photon flux at that wavelength, then integrating from 300 to 800 nm (Table 5). The YPF model adjusts PPFD based on the likelihood that a photon of a given wavelength will be absorbed and the likelihood that the energy will be used for photosynthesis once absorbed.

Finally, photostationary state of phytochrome (PSS), an estimate of active phytochrome as a portion of total phytochrome, was calculated using

(1) $$PSS=(\overset{800}{\underset{300}{\sum }}N_{\lambda }\sigma_{r{\;}_{\lambda }})/{\left(\overset{800}{\underset{300}{\sum }}N_{\lambda }\sigma_{r{\;}_{\lambda }}+\overset{800}{\underset{300}{\sum }}N_{\lambda }\sigma_{fr{\;}_{\lambda }}\right)}^{\;}$$

as reported in Table 2 [9].

Equation (1) gives *PSS* where *N* is incident photon flux at a given wavelength (*λ*), *σ_r_* is the photochemical cross section of P_r_ (the red-absorbing, inactive form of phytochrome) at *λ*, and *σ_fr_* is the photochemical cross section of P_fr_ (the far-red-absorbing, active form of phytochrome) at *λ*.

Photosynthesis was measured in two ways. First, using a LI-6400 with a clear-top chamber (LI-COR Biosciences, Lincoln, NE, USA), net photosynthesis (A) and stomatal conductance (g_s_) were measured at an ambient CO_2_ concentration (C_a_) of 400 ppm and a leaf temperature of 25 °C, illuminated by treatment light spectra at ambient intensity.

Second, A vs. C_c_ response curves, the net photosynthesis rate obtained under varying concentrations of CO_2_ in the chloroplast (C_c_) under saturating light, were measured using the LI-6400 portable photosynthesis system with a 6400-40 leaf chamber fluorometer attachment (LI-COR Biosciences, Lincoln, NE, USA) in order to gain insight about possible molecular adaptations to the light environment. Measurements were taken at external CO_2_ concentrations of 400, 300, 250, 200, 150, 100, 50, 400, 500, 600, 850, and 1000 ppm in that order. Following initial fluorescence measurements, the plants had half an hour to adapt to light at 1000 μmol m^−2^ s^−1^. Each CO_2_ concentration was held for two to four minutes at a flowrate of 300 μmol s^−1^ with leaf temperature set to 25 °C while the plant was at room temperature (23 to 26 °C). The first true leaf, unshaded by neighboring leaves, was measured.

Plants were first dark adapted for half an hour before initial measurements. Fluorescence measurements were taken on the dark-adapted leaves, before acclimating to the light for a half hour. The minimum chlorophyll fluorescence for dark-adapted leaves (F_o_), maximum light- and dark-adapted chlorophyll fluorescence (F_m_’ and F_m_, respectively), and steady state light-adapted chlorophyll fluorescence (F’) were measured. The maximum quantum efficiency of PSII (F_v_/F_m_), the relative PSII operating efficiency (ΦPSII), the coefficient of photochemical quenching (q_p_), the quantum yield of non-light-induced nonphotochemical quenching (ΦNPQ), and the quantum yield of light-induced nonphotochemical quenching (ΦNO) were calculated according to [37]. The fraction of oxidized plastoquinone, q_L_, was calculated according to [55]. Due to the difficulties of measuring F_o_’, the minimal fluorescence of a light-adapted leaf, it was calculated using the equation F_o_’ = F_o_/[(F_v_/F_m_) + (F_o_/F_m_’)] where F_o_ is the minimal fluorescence of a dark-adapted leaf, F_m_ is the maximal fluorescence from a dark-adapted leaf, F_m_’ is the maximal fluorescence from a light-adapted leaf, and F_v_ is the difference between F_m_ and F_o_ [40].

A vs. C_c_ curve fitting was done using SAS Studio 3.8 software via the NLIN procedure, a procedure for fitting nonlinear models, using Equations (2)–(4) [56,57]. Typically, these model fittings involve 3 segments representing photosynthesis as limited either by the maximum ribulose-1,5-bisphosphate (RuBP) carboxylation rate Equation (2), the RuBP regeneration rate Equation (3), or the triose phosphate utilization (TPU) rate. However, our data suggest that TPU was not a limiting factor and so we fitted to only the Rubisco-limiting (Equation (2)) and the RuBP-limiting curves (Equation (3)). The equation for calculating the concentration of CO_2_ at Rubisco, C_c_, has also been included (Equation (4)).

(2) $$A=V_{cmax}\left[\frac{C_{c}-\Gamma^{*}}{C_{c}+K_{c}\left(1+\frac{O}{K_{o}}\right)}\right]-R_{d}$$

(3) $$A=J\left[\frac{C_{c}-\Gamma^{*}}{4C_{c}+8\Gamma^{*}}\right]-R_{d}$$

(4) $$C_{c}=C_{i}-\frac{A}{g_{m}P_{atm}}$$

where *C_i_* is the intercellular concentration of CO_2_, *C_c_* is the concentration of CO_2_ at Rubisco, *A* is net CO_2_ assimilation, *V_cmax_* is maximum carboxylation rate of Rubisco, Γ^*^ is the point at which oxygenation is twice the rate of carboxylation (CO_2_ uptake equals CO_2_ photorespiratory release), *K_o_* is the inhibition constant of Rubisco for oxygen, *K_c_* is the Michaelis–Menten constant of Rubisco for CO_2_, *O* is the partial pressure of O_2_ at Rubisco, *R_d_* is non-photorespiratory CO_2_ release, *J* is the rate of electron transport, *P_atm_* is atmospheric pressure, and *g_m_* is mesophyll conductance.

Due to the difficulty of accurately determining g_m_ due to the method of data collection and initial fittings determining that g_m_ did not significantly differ between any treatments, the overall average value of 2.12 µmol m^−2^ s^−1^ Pa^−1^ was used [58]. Likewise, since estimates of R_d_ did not significantly differ between treatments, an average value of 2.71 µmol CO_2_ m^−2^ s^−1^ was used.

All plants’ shoots were severed at the substrate surface and weighed for fresh weight, separated into leaf blades and all other material (stem, petioles, cotyledons, and leaves < 2 cm^2^), oven dried for 72 h at 60 °C, and weighed to obtain dry weights. Stem diameter was measured with an electronic caliper just below the cotyledons with the caliper arm held parallel to the cotyledons to give a consistent measurement for seedlings with non-circular stem cross-sections. Stem height was measured from the point at which the shoot was severed to the base of the apical meristem to the nearest millimeter. The two largest leaves on each plant had length, width, petiole length, and leaf blade area measured. Additionally, total leaf area was measured using a LI-COR 3100 leaf area meter (LI-COR Biosciences, Lincoln, NE, USA). Finally, stomatal density was measured by taking a 1 cm by 2 cm section of leaf tissue adjacent to the midrib approximately halfway between leaf tip and leaf blade base and applying clear nail polish [59].

All means separations were determined using SAS Studio software 3.8 (SAS Institute Inc., Cary, NC, USA). Data from the two replications were treated as separate blocks with means separation analyzed by a Tukey–Kramer HSD (*p* = 0.05).

## 5. Conclusions

Plants adapt to light signals by adjusting photosystem stoichiometry. In monochromatic red light, this reduces photosynthetic capacity of the plant under broad spectra and saturating light conditions. However, this stoichiometric imbalance is not seen in spectra containing blue light and is partially remediated by spectra containing green light. Despite this observance under saturating light conditions, monochromatic green light had lower net photosynthesis rates than monochromatic red light under ambient conditions. Nevertheless, other factors, such as morphological adaptations like height, leaf area, and SLW seem to drive biomass accumulation as much or more than net photosynthesis per unit leaf area, given that plants grown under monochromatic blue light were tied for lowest ambient net photosynthetic rate with monochromatic green light, but the B treatment produced more massive plants than the GB, R, and RB treatments which all had higher net photosynthesis under ambient conditions than the B treatment plants.

## Figures and Tables

**Table 1:** Maximum carboxylation rate of Rubisco (Vcmax) and potential rate of photosynthetic electron transport (J) estimated for each light treatment. Different letters indicate significant differences (*p* ≤ 0.05; *n* = 3 or 4).

Treatment	J	V_cmax_
	µmol m^−2^ s^−1^	µmol m^−2^ s^−1^
B	132.0 ± 5.3 c	87.0 ± 4.6 b
G	83.4 ± 1.2 d	53.6 ± 1.2 c
GB	155.5 ± 4.3 ab	102.1 ± 3.6 ab
R	54.3 ± 0.7 e	32.2 ± 0.8 d
RB	157.3 ± 2.8 a	103.0 ± 2.3 a
RG	82.1 ± 1.3 d	49.0 ± 1.2 c
RGB	140.5 ± 1.6 bc	93.2 ± 1.2 b
RGB + FR	137.7 ± 2.7 c	101.9 ± 2.1 a

**Table 2:** Maximum quantum efficiency of PSII (Fv/Fm), relative PSII operating efficiency (ΦPSII), coefficient of photochemical quenching (qp), the quantum yield of non-light-induced nonphotochemical quenching (ΦNPQ), the quantum yield of light-induced nonphotochemical quenching (ΦNO), and the fraction of oxidized plastoquinone (q_L_) calculated using measurements under saturating (1000 µmol photons m^−2^ s^−1^) 90% red, 10% blue light. Different letters indicate significant differences (*p* ≤ 0.05; *n* = 3 or 4).

Treatment	ΦPSII	F_v_/F_m_	ΦNPQ	ΦNO	q_P_	q_L_
B	0.26 ± 0.01 b	0.81 ± 0.01 a	0.48 ± 0.01 c	0.26 ± 0.00 f	0.43 ± 0.01 b	0.23 ± 0.01 ab
G	0.14 ± 0.00 c	0.80 ± 0.00 a	0.54 ± 0.00 a	0.32 ± 0.00 c	0.24 ± 0.01 c	0.12 ± 0.00 d
GB	0.29 ± 0.01 a	0.82 ± 0.00 a	0.44 ± 0.01 e	0.26 ± 0.00 f	0.46 ± 0.01 a	0.24 ± 0.01 a
R	0.09 ± 0.00 e	0.79 ± 0.01 a	0.53 ± 0.00 a	0.38 ± 0.00 a	0.14 ± 0.00 e	0.06 ± 0.00 f
RB	0.26 ± 0.01 b	0.82 ± 0.01 a	0.47 ± 0.01 cd	0.26 ± 0.00 f	0.42 ± 0.01 b	0.22 ± 0.01 bc
RG	0.12 ± 0.00 d	0.80 ± 0.01 a	0.51 ± 0.00 b	0.37 ± 0.00 b	0.19 ± 0.01 d	0.08 ± 0.00 e
RGB	0.27 ± 0.01 b	0.82 ± 0.00 a	0.43 ± 0.01 e	0.29 ± 0.00 d	0.42 ± 0.01 b	0.20 ± 0.01 c
RGB + FR	0.27 ± 0.01 b	0.81 ± 0.01 a	0.45 ± 0.01 de	0.28 ± 0.00 e	0.43 ± 0.01 b	0.23 ± 0.01 b

**Table 3:** Stomatal density, abaxial (AB) to adaxial (AD) stomatal density ratio, intrinsic water use efficiency, and water content of cucumber.

Treatment	Abaxial	Adaxial	AB:AD	iWUE	Water Content
	Stomata/mm^2^	Stomata/mm^2^		µmol CO_2_ mol^−1^ H_2_O	g H_2_O 100 g^−1^ FW
B	402 ± 22 bcd	243 ± 16 bc	1.66 ± 0.11 b	12.5 ± 1.4 d	92.1 ± 0.2 a
G	378 ± 26 cde	153 ± 20 de	2.67 ± 0.13 a	37.1 ± 1.5 b	92.4 ± 0.2 a
GB	507 ± 25 a	349 ± 18 a	1.55 ± 0.12 b	17.9 ± 1.4 cd	91.0 ± 0.2 b
R	295 ± 25 e	116 ± 19 e	2.76 ± 0.13 a	37.4 ± 1.4 b	92.7 ± 0.4 a
RB	490 ± 22 ab	254 ± 16 b	1.99 ± 0.11 b	22.6 ± 1.4 c	90.6 ± 0.1 b
RG	312 ± 24 de	175 ± 18 cde	1.82 ± 0.12 b	47.0 ± 1.3 a	92.0 ± 0.3 a
RGB	486 ± 25 abc	287 ± 19 ab	1.73 ± 0.13 b	20.9 ± 1.4 c	90.6 ± 0.2 b
RGB + FR	363 ± 21 de	224 ± 16 bcd	1.66 ± 0.11 b	23.0 ± 1.4 c	92.3 ± 0.3 a

**Table 4:** Color breakdown for light treatment spectra as a percentage of total photosynthetic photon flux density (PPFD). Wavelength ranges for the traditional method indicate the typically defined range for each color, while the wavelength range for the bar method indicates the range in which >99% of the light is emitted from a given bar color.

Traditional method	**Treatment**	**Red** **(600–700 nm)**	**Green** **(500–600 nm)**	**Blue** **(400–500 nm)**
	B	0	1	99
	G	0	93	6
	GB	0	37	63
	R	100	0	0
	RB	47	1	52
	RG	65	31	3
	RGB	38	22	40
	RGB + FR	40	20	40
Bar method	**Treatment**	**Red** **(623–684 nm)**	**Green** **(486–582 nm)**	**Blue** **(432–500 nm)**
	B	0	0	100
	G	0	100	0
	GB	0	38	62
	R	100	0	0
	RB	47	0	53
	RG	65	35	0
	RGB	38	23	39
	RGB + FR	40	21	39

**Table 5:** Yield photon flux (YPF) and photostationary state of phytochrome (PSS) for each light treatment.

Treatment	YPF	PSS
	µmol m^−2^ s^−1^	P_fr_:P_total_
B	88	0.51
G	94	0.83
GB	90	0.62
R	114	0.89
RB	98	0.86
RG	106	0.88
RGB	102	0.86
RGB + FR	104	0.76

## Data Availability

Data are available upon request.
